# Antimicrobial, Anti-Inflammatory, Antiparasitic, and Cytotoxic Activities of *Laennecia confusa*

**DOI:** 10.1100/2012/263572

**Published:** 2012-04-30

**Authors:** María G. Martínez Ruiz, Melissa Richard-Greenblatt, Zaida N. Juárez, Yossef Av-Gay, Horacio Bach, Luis R. Hernández

**Affiliations:** ^1^Departamento de Ciencias Químico-Biológicas, Universidad de las Américas Puebla, Santa Catarina Mártir s/n, 72820 Cholula, PUE, Mexico; ^2^Division of Infectious Diseases, Department of Medicine, University of British Columbia, Vancouver, BC, Canada V5Z 3J5; ^3^Departamento de Ciencias Biológicas, Facultad Biotecnoambiental, Universidad Popular Autónoma del Estado de Puebla, 72410, Puebla, PUE, Mexico

## Abstract

The current paper investigated the potential benefit of the traditional Mexican medicinal plant *Laennecia confusa* (Cronquist) G. L. Nesom (Asteraceae). Fractions from the hexane, chloroform, methanol, and aqueous extracts were analyzed for antibacterial, antifungal, anti-inflammatory, and antiparasitic activities. The antimicrobial activity of the extracts and fractions was assessed on bacterial and fungal strains, in addition to the protozoa *Leishmania donovani*, using a microdilution assay. The propensity of the plant's compounds to produce adverse effects on human health was also evaluated using propidium iodine to identify damage to human macrophages. The anti-inflammatory activity of the extracts and fractions was investigated by measuring the secretion of interleukin-6. Chemical analyses demonstrated the presence of flavonoids, cyanogenic and cardiotonic glycosides, saponins, sesquiterpene lactones, and triterpenes in the chloroform extract. A number of extracts and fractions show antibacterial activity. Of particular interest is antibacterial activity against *Staphylococcus aureus* and its relative methicillin-resistant strain, MRSA. Hexanic and chloroformic fractions also exhibit antifungal activity and two extracts and the fraction CE 2 antiparasitic activity against *Leishmania donovani*. All bioactive extracts and fractions assayed were also found to be cytotoxic to macrophages. In addition, the hexane and methane extracts show anti-inflammatory activity by suppressing the secretion of interleukine-6.

## 1. Introduction

Ethnomedicine can be documented as far back as the Middle Paleolithic age, some 60,000 years ago [1]. Several years ago, the WHO Traditional Medicine Programme identified a total of 122 plant-derived compounds employed clinically, demonstrating the value of plants in drug discovery [1]. Still much of the vast body of knowledge in traditional medicine remains unexplored in regards to drug development.

The genus *Conyza* (Asteraceae) is comprised of approximately 400 species [2], and several species are known for their use in traditional medicine. *Conyza blinii* is used in Chinese folk medicine to treat gastroenteritis, chronic bronchitis, and other inflammatory diseases [3]. *C. canadensis, C. albida*, *C. bonariensis,* and *C. dioscoridis *also exhibit antiinflammatory, antidiarrhoeal, antimicrobial, antiparasitic, antinociceptive, antioxidant, and expectorant activities as well as antiaggregatory effects on blood platelets and the inhibition of catecholamine secretion [2, 4–11].

Currently, there are a number of bioactive compounds, namely, alkenynes, terpenoids, terpenes, saponins, flavonoids, sterols, phenylpropanoyl esters, lactones, tannins, and coumarins, which have been isolated from the *Conyza* genus [3–5, 8, 9, 11–16].

Several epithets of the *Conyza* genus have been recently transferred to the genus *Laennecia* [15]. One such a transfer is *Laennecia confusa* (Cronquist) G. L. Nesom [17], a native Mexican plant found between the states of Chihuahua and Chiapas. It grows preferentially at a range of 2550–3200 m above sea level in conifer and *Quercus* forests [18].* L. confusa* is prepared as a stem infusion and used in Mexican folk medicine as a sedative and treatment for alcoholic addiction [19]. Because of the traditional use of *L. confusa* and the taxonomically close resemblance to other *Conyza *species exhibiting other bioactivities, the pharmacological potential of the plant's extracts was investigated. In the current study, we evaluated the presence of antimicrobial components in extracts and fractions obtained from the stems of* L. confusa*. In addition, we tested its anti-inflammatory, antiparasitic, and cytotoxic activities to evaluate and expand its potential use.

## 2. Materials and Methods

### 2.1. Plant Material

Commercially available* L. confusa* was purchased from a herb shop at Puebla, México. Plants were collected at Atlixco, Puebla, México, in January 2008, and were analyzed by Carlos Marín Martínez at the Centro Botánico de Plantas Medicinales, Puebla, México.

### 2.2. Preparation of Plant Extracts

**Figure 1:**
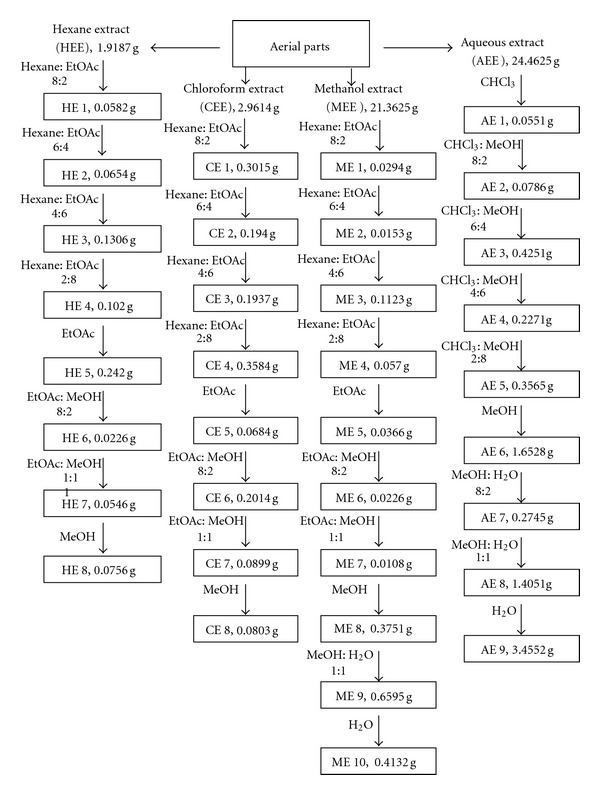
Diagram showing the fractionation of the hexane, chloroform, methanol, and aqueous extracts extracted from the aerial parts of *Laennecia confusa*. Ratios of the combined solvents are expressed in volume/volume.

A total of 200 g of air-dried aerial parts of *L. confusa* were sequentially extracted with *n*-hexane (hexane), chloroform, and methanol, after macerating the material in each solvent in three rounds for 48 h at room temperature. To simulate a herb infusion (according to the traditional preparation), an aqueous extraction was carried out by soaking 200 g of the same plant material in boiled distilled water for 2 h. Following filtering, solvent volatilization, and lyophilization (for aqueous extract), the extracts were chromatographed on silica gel (70–230 mesh) using approximately 300 mL of pure or combined solvents according to Figure 1. Fractions were dried in a vacuum using a Rotavapor and stock solutions were prepared by dissolving 20 mg of each fraction in 100 *μ*L DMSO (or equivalent to obtain the same final amount), following sonication (Branson 3210) for 60 min at 30°C until the material was completely dissolved.

### 2.3. Chemical Analysis of the Extracts

*Triterpenes*, *Saponins*, *Flavonoids*, *and Tannins*. The presence of triterpenes was determined by dissolving 10 mg of the CEE in 1 mL chloroform. The solution was supplemented with 1 mL of acetic anhydride and 2 drops of H_2_SO_4_. The appearance of red, pink, green, purple, or blue coloration in the interface indicates the presence of triterpenes [20]. Two different methods were used to determine the presence of saponins. In the first one, 10 mg of CEE was mixed with hot water and the mixture was shaken for 30 sec. The formation of stable foam indicates the presence of saponins. In the second method, a drop of Rosenthaler reagent (1 g of potassium arsenate dissolved in 100 g H_2_SO_4_) was added to 10 mg CEE. The appearance of violet coloration indicates the presence of saponins [20]. The presence of flavonoids was evaluated using two methods. The first method was performed by adding 100 mg of zinc or magnesium powder to 10 mg of CEE and three drops of HCl. The appearance of red coloration indicates the presence of flavonoids [20]. In the second method, three drops of NaOH were added to 10 mg of CEE, and the appearance of yellow or orange colour indicates the presence of flavonoids [20]. Tannins were determined by preparing a chloroformic solution of 10 mg of CEE. The mixture was filtrated and separated in two portions. The presence of tannins was evaluated by the formation of a precipitate by adding either 100 mg FeCl_3_ or gelatine reagent ((1% gelatine (*w/v*)) [20].

*Cardiotonic Glucoside*, *Sesquiterpene Lactones*, *Alkaloids*, *Cyanogenic Glycosides, and Anthraquinones*. Two methods were used to determine the presence of cardiotonic glucosides and sesquiterpene lactones. In the first method, 10 mg of CEE was dissolved in 1 mL chloroform. Three drops of Baljet reagent (1% ethanolic solution of picric acid and 10% aqueous solution of NaOH) were added [20]. Formation of red or orange coloration indicates the presence of these compounds. In the second method, 10 mg of CEE was dissolved in 1 mL chloroform. Three drops of pyridine, one drop of 5% sodium nitroprusside, and three drops of 10% aqueous solution of NaOH were added sequentially. Red coloration indicates the presence of cardiotonic glucosides and sesquiterpene lactones. Alkaloids were determined by preparing a solution of 10 mg of CEE dissolved in 5% HCl. The reagents of Mayer, Dragendorff, and Wagner were used to determine the presence of these compounds [20]. Cyanogenic glycosides were measured by dissolving 10 mg of CEE in 5 drops of chloroform in a 5 mL glass tube. A piece of filter paper was impregnated with Grignard reagent (100 mg Na_2_CO_3_, 10 mg picric acid in 10 mL distilled water) and placed on top of the tube. The appearance of a pink or red color after heating the solution at 35°C indicates the presence of these compounds [20].

*Anthraquinones and Steroids*. The Borntrager reaction was used to detect the presence of anthraquinones. 300 mg of the plant was pulverized for 5 min in presence of 10 mL of 0.5 N KOH and 1 mL of 6% H_2_O_2_ and filtrated. The filtrate was acidified with 10 drops of acetic acid and extracted with 10 mL benzene. The organic phase was separated and mixed with 2.5 mL of NH_4_OH. A red color in the alkaline layer indicates the presence of anthraquinones [20]. The presence of steroids was detected by the appearance of green, blue, pink, or purple colors after mixing 300 mg of the pulverized plant with 0.5 mL acetic anhydride and 2 drops of H_2_SO_4_ [20].

### 2.4. Strains and Culture Media

The bacterial strains used in this study included the Gram-negative strains *Acinetobacter baumannii* (ATCC BAA-747), *Escherichia coli* (ATCC 25922), *Pseudomonas aeruginosa* (ATCC 14210), and *Klebsiella pneumoniae *(ATCC 8044). *Bacillus subtilis* (ATCC 6633), *Mycobacterium smegmatis* mc^2^155 (ATCC 700084), *Staphylococcus aureus* (ATCC 25923), Methicillin-Resistant *Staphylococcus aureus* (MRSA) (ATCC 700698), and *Streptococcus pyogenes* (ATCC 51878) were used as representatives of Gram-positive bacteria. *Aspergillus fumigatus* (ATCC 1022), *Candida albicans* (provided by Vancouver General Hospital, BC, Canada), *Cryptococcus neoformans* var. *grubii* (kindly provided by Dr. Karen Bartlet, University of British Columbia, BC, Canada), and *Trichophyton rubrum* (ATCC 18758) were tested as representatives of pathogenic fungi. The parasite *Leishmania donovani* Sudan strain 2S was assessed for antiparasitic activity of the extracts and was generously provided by Dr. Neil Reiner (University of British Columbia, Vancouver, BC, Canada).

Bacterial strains were cultured in Mueller-Hinton broth (B&D) except for* Mycobacterium smegmatis, *which was cultured in Trypticase Soy broth (B&D). Bacterial stocks were maintained on the same broth supplemented with 1.5% agar (B&D) at 4°C. All the bacterial strains were cultured at 37°C. Fungi were cultured in Sabouraud broth (B&D), and the antifungal activity against filamentous fungi was assessed from spores. Specifically, spores of *A. fumigatus* and* T. rubrum* were incubated at 28°C until sporulation. Spores were harvested by carefully rubbing the top of sporulated colonies in 2 mL Sabouraud broth containing 10% glycerol. Spores were aliquoted and kept at −20°C. For *C. albicans* and *C. neoformans*, the same protocol used for bacterial strains was followed, but using Sabouraud broth *L. donovani *promastigotes were cultured in medium M199 supplemented with 10% fetal bovine serum (Gibco), 1% penicillin and streptomycin, 20 mmol/l Hepes (Stem Cell Technologies), 6 *μ*g/mL hemin, 2 mmol/L L-glutamine, 10 *μ*g/mL folic acid, and 100 *μ*mol/L adenosine at 26°C in a EchoTherm Chilling Incubator (Torrey Pines Scientific, San Marcos, CA, USA). Every third day the organisms were split 1 : 10 into fresh medium.

### 2.5. Antimicrobial and Antiparasitic Assays

A microdilution assay was used to assess the antimicrobial activities using a 96-well plate and according to published protocols [21]. Bacterial strains were grown overnight by shaking at 37°C. The next day, the inoculum density for the bacterial strains was adjusted to 0.5 on the McFarland scale [22] with an optical density of 0.080–0.100 at 625 nm. Extracts and fractions at concentrations of 2, 20, 30, 50, 100, 200, 500, 1000, and 2000 *μ*g/mL were evaluated in a final volume of 150 *μ*L/well. Minimal inhibitory concentrations (MICs) were determined by incubating the organisms in 96-well microplates for 24 h at 37°C. Endpoints were determined when no turbidity in the well was observed. DMSO and untreated inoculum were used as negative controls, while gentamicin was used as a positive control. Antifungal properties were evaluated in RPMI 1640 medium supplemented with L-glutamine without NaHCO_3_ (Gibco). Fungal strains were grown at 28°C for 24 h using the same 96-well format, and in the case of *T. rubrum *and *A. fumigatus*, plates were incubated at the same temperature but for a period of 72 h. DMSO and untreated inoculum were used as negative controls, while fluconazole and amphotericin B were used as positive controls.

The evaluation of antiparasitic activity was performed in 24-well flat bottom plates containing 1 × 10^6^ promastigotes/well. Compounds were evaluated at a final concentration of 0.02, 0.2, and 2 mg/mL. Untreated parasites and DMSO were used as negative controls. Motility and number of parasites were registered at 24, 48, and 72 h, after staining the sample with 1% Trypan blue solution. Only bioactive extracts and fractions are shown.

### 2.6. Cytotoxicity Assays

The human-derived monocytic cell line THP-1 (ATCC 202) was cultured in RPMI 1640 (Hyclone) supplemented with 5% fetal calf serum (FCS) (Hyclone), and 2 mM L-glutamine (Stem Cell Technologies). THP-1 cells were dispensed at a concentration of 3 × 10^5^/well in a 96-well plate and incubated with compound concentrations that showed antibacterial or antifungal activity. Plates were placed at 37°C in a humidified atmosphere supplemented with 5% CO_2_ for 24 h. Nontreated THP-1 cells or DMSO were used as negative controls. THP-1 cells exposed to 5% hydrogen peroxide were used as a positive control. Propidium iodide (PI) was used to evaluate cell damage according to published procedures [23]. Results are presented as percentage of the distribution area as calculated by the Flow Cytometer software.

### 2.7. Anti-Inflammatory Assay

The anti-inflammatory activities of the extracts were studied *in vitro* by measuring the secretion of the proinflammatory interleukin 6 (IL-6) from THP-1 cells. THP-1 cells were seeded at a concentration of 3 × 10^5^/well in a 96-well plate. Monocytes were cultured as described previously. Before the induction of an inflammatory process, cells were incubated with 20 *μ*g/mL HEE, CEE, MEE, or AEE for 5 h at 37°C supplemented with 5% CO_2_. DMSO was used as a negative control. Next, an inflammatory process was induced by addition of 50 *μ*L lipopolysaccharide (LPS) (Sigma) at a final concentration of 100 ng/mL after washing the cells with fresh RPMI medium (x3). After 3 h of incubation, the supernatants were transferred to a new 96-well plate and stored at −20°C for further processing. A sandwich ELISA was used to evaluate the secretion of IL-6 according to published protocols [24]. Human anti-IL-6 antibody (RD Systems) was used as primary antibody, while a mixture of biotinylated human anti-IL-6 diluted 1 : 6000 (RD Systems) and goat HRP-anti-biotin diluted 1 : 250 (RD Systems) was used as a secondary antibody. 3,3′,5,5′ tetramethylbenzidine was used as a developer, and reactions were stopped with 25 *μ*L of 1 M sulphuric acid. Plates were read immediately in an ELISA plate reader using a 450 nm filter. The commercial available steroids prednisolone and dexamethasone (Sigma) were used as positive controls. Nontreated macrophages were used as a negative control. Experiments were performed in triplicate. 

### 2.8. Statistical Analysis

A Mann-Whitney test was used for statistical analysis. A *P* value < 0.05 was considered significant.

## 3. Results

### 3.1. Chemical Constituents of the Extracts

The hexane, chloroform, methanol, and aqueous extracts (HEE, CEE, MEE, and AEE, resp.) yielded 1.92 g (0.96%), 2.96 g (1.48%), 21.36 g (10.68%), and 24.46 g (12.23%) of residue, respectively. A total of 71 fractions corresponding to the hexane, chloroform, methanol, and aqueous extracts were collected and combined according to their TLC profile. Eight hexanic fractions, eight chloroformic fractions, ten methanolic fractions, and nine aqueous fractions were obtained. The corresponding weight of each fraction is listed in Figure 1. Chemical analyses of the CEE show the presence of flavonoids, cyanogenic and cardiotonic glycosides, saponins, sesquiterpene lactones, and triterpenes (data not shown). No alkaloids, anthraquinones, steroids, or tannins were detected in the assayed extract.

### 3.2. Antibacterial Activity

*L. confusa* HEE, CEE, and MEE were analyzed for their antibacterial activity against several Gram-negative and Gram-positive strains. The chloroformic and methanolic fractions CE 4 and ME 3 inhibited the growth of *K. pneumoniae* at concentrations of 1000 *μ*g/mL, whereas the methanolic fraction ME 4 inhibited the growth of both *E. coli* and *P. aeruginosa* at the same concentration (Table 1). No activity against the Gram-negative strains was observed when any of the extracts were evaluated. Growth inhibition was also observed in two of the Gram-positive bacteria, MRSA and *S. aureus*. Two hexanic (HE 1, HE 3), two chloroformic (CE 3, CE 4), and three methanolic (ME 3, ME 4, ME 5) fractions were determined to possess antibacterial activity against MRSA at final concentrations of 200 or 1000 *μ*g/mL (Table 1), while *S. aureus* was inhibited by the same hexanic fractions and the ME 3 fraction at the same concentrations. Growth inhibition of *S. aureus* was also observed by the CEE extract at a concentration of 1000 *μ*g/mL (Table 1), suggesting the presence of antibacterial compound(s) against staphylococcal strains. No bioactivity was observed in the solvent control.

### 3.3. Antifungal Activity

Three out of four fungal strains assayed were inhibited by at least one of the fractions analyzed. Three hexanic fractions (HE 3, HE 4, HE 8) and the same number of chloroformic (CE 1, CE 2, CE 3) fractions show activity against *Candida albicans* at concentrations of 1000 *μ*g/mL, while the same hexanic fractions and the chloroformic fraction CE 3 inhibited the grow of *C. neoformans* at concentrations of 200 or 1000 *μ*g/mL. Spore growth inhibition of *T. rubrum* occurred at MICs varying from 30 to 100 *μ*g/mL of the hexanic fractions (HE 3, HE 4, HE 8) (Table 1). No bioactivity was observed in the solvent control. No antifungal activities were detected against *A. fumigatus*.

### 3.4. Antiparasitic Activity

**Figure 2:**
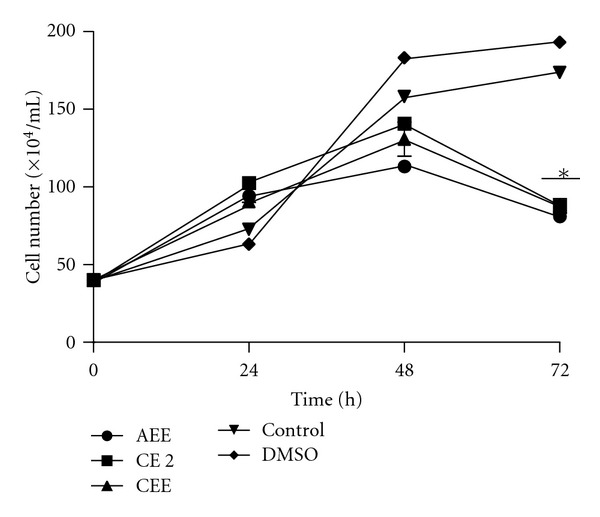
Antiparasitic activity exhibited by fractions of *Laennecia confusa*. *Leishmania donovani* promastigote growth inhibition was evaluated after incubation of the parasites with the compounds. Untreated promastigotes and DMSO were used as negative controls. Control: untreated promastigotes. Shown is the mean ± SD of three independent experiments. *P value < 0.0001.

A reduction in growth of *L. donovani* in comparison to the controls from the aqueous and chloroform extracts and the chloroformic fraction CE 2 was observed in the antiparasitic assay. A decrease of approximately 50% in the number of parasites was measured after 72 h after exposure of the tested compounds (Figure 2). IC_50_ values of 20 *μ*g/mL were calculated for both aqueous and chloroform extracts, whereas an IC_50_ of 200 *μ*g/mL was measured for the chloroformic fraction CE 2 (Figure 2). 

### 3.5. Cytotoxic Activity

**Figure 3:**
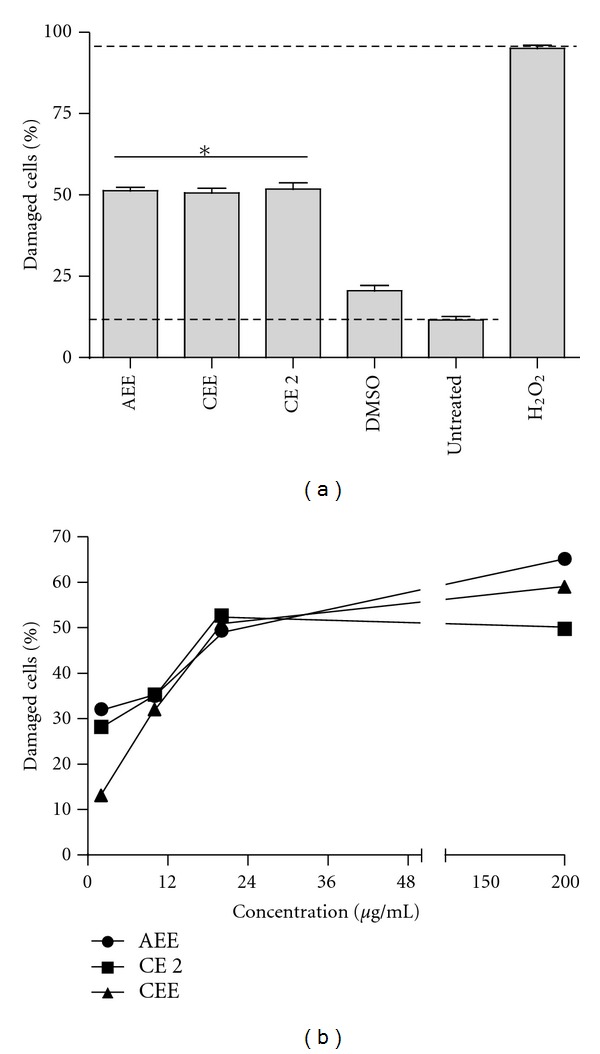
Cytotoxic effects of compounds from *Laennecia confusa*. (a) THP-1 cells were used to assess the cytotoxic effects of bioactive compounds using propidium iodine staining and the obtained values were used to calculate the IC_50_ (b). Dashed lines represent treatment with 5% H_2_O_2_ (upper line) (positive control) and untreated cells (lower line) (negative control). Shown is the mean ± SD of three independent experiments. CE is a chloroformic fraction, and AEE and CEE are aqueous and chloroform extracts, respectively. *P value < 0.0001.

The extracts and fractions demonstrating antiparasitic activity in the previous assay were incubated with the human-derived monocyte THP-1 cells to determine their cytotoxic effects. Following 24 h exposure, all of the tested compounds exhibited toxicity to macrophages (Figure 3(a)) with calculated IC_50_ (±SD) values of 24.8 ± 1.89, 25 ± 2.35, and 24.2 ± 1.23 *μ*g/mL for the AEE and CEE and the chloroformic fraction CE 2, respectively (Figure 3(b)). 

### 3.6. Anti-Inflammatory Activity

**Figure 4:**
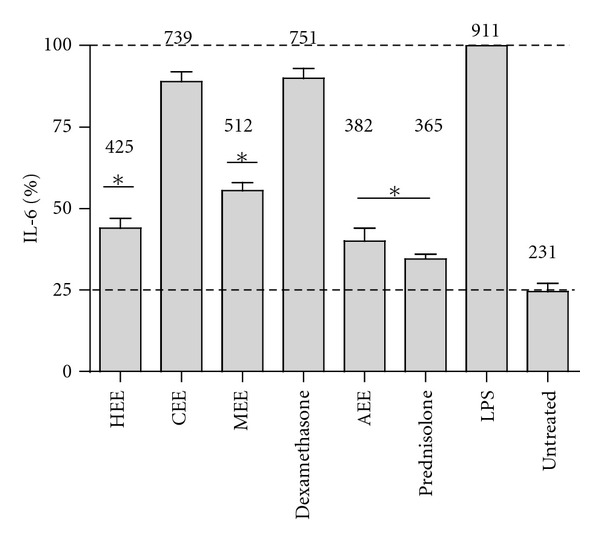
Anti-inflammatory activity of extracts and fractions from *Laennecia confusa*. An inflammatory process was elicited by exposing macrophages to LPS. Crude extracts were exposed to macrophages before the addition of LPS. The anti-inflammatory process was evaluated by measuring the secretion of IL-6 into the supernatant. Shown is the mean ± SD of three independent experiments. Numbers on the bars indicate the concentration of IL-6 in pg/mL. HEE, CEE, MEE, and AEE are hexane, chloroform, methanol, and aqueous extracts, respectively. *P value < 0.0001.

Human-derived macrophages were exposed to each of the extracts and then an inflammatory response was induced with LPS. To evaluate the inflammatory response, the secretion of the pro- and anti-inflammatory IL-6 cytokine was measured in the supernatant of the cultures. A concentration of 580 pg/mL of IL-6 was measured in the supernatant when LPS was added to the macrophages, while levels of 233 and 251 pg/mL were measured when these cells were pretreated with HEE and MEE, respectively (Figure 4). These values represent a reduction of approximately 60% in the secretion of IL-6. Similar results were measured when macrophages were exposed to prednisolone or dexamethasone, suggesting both extracts contains anti-inflammatory compounds. No effects were observed when either the CEE or AEE was supplemented to the cultures.

## 4. Discussion

Several species from the *Conyza* (*Laennecia*) genus are used in traditional medicine for the treatment of inflammatory and infectious ailments [8]. Previous studies [3, 5, 10] have confirmed bioactive properties for specific *Conyza* species, triggering further interest in *Conyza*-derived drug discovery. The chemical investigation of *L. confusa* revealed a close chemical resemblance to several *Conyza* (*Laennecia*) species leading to the exploration of the plant's antimicrobial, antiparasitic, and anti-inflammatory properties in this study.

The current study shows that extracts and fractions from *L. confusa* possess antimicrobial activity against both Gram-negative and Gram-positive organisms. Particularly, the majority of antibacterial is confined to *S. aureus* and its methicillin-resistant counterpart. These findings are of particular importance due to increasing resistance of MRSAs to beta-lactam antibiotics rendering the infection difficult to treat using current methods [25]. *L. confusa* fractions also show antibacterial activity against the Gram-negative strains *E. coli*, *K. pneumonia*, and *P. aeruginosa*. Our results are in line with those reported for other *Conyza* species. For instance, the compounds conyzolide, conyzoflavone, and 8*R*, 9*R*-dihydroxymatricarine methyl ester isolated from *C. canadensis* were active against *S. aureus* and *E. coli* [9, 11, 26].

A number of fractions and extracts were also found to have antifungal activity against yeast-like strains *C. albicans, *and *C. neoformans*, and against the filamentous* T. rubrum*. The flavones, glycolides, glycosides, steroids and triterpenoids isolated from *Conzya *species' extracts are reported to possess comparable antifungal properties against these and additional fungi such as *Fusarium solani* and *Microsporum gypsum* [9, 26].

Antiprotozoal activity of the fractions and extracts was also assessed against *L. donovani*. A fraction of the CEE, CE 2, and the CEE and AEE display strong inhibitory effects on growth following 48 h of incubation. Similar values of IC_50_ ranging between 2 and 70 *μ*g/mL inhibiting the growth of *Plasmodium falciparum* were reported using extracts of *C. albida*, *C. podocephala*, and *C. scabrida* [6]. Moreover, extracts from *C. filaginoides* showed IC_50_ values ranging between 14–119 and 15–178 *μ*g/mL against *Entamoeba histolytica*, and* Giardia lamblia*, respectively [3].

The AEE, CEE, and the chloroformic fraction CE 2 demonstrated that antiparasitic activity was further analyzed for their cytotoxicity against macrophages. Although the PI assay identified all of the samples analyzed as cytotoxic to macrophages, these extracts can still be developed for topical applications, such as antimicrobial and antileishmanial formulations. Other studies reported that an AEE of *Conyza bonariensis* also showed cytotoxicity with the brine shrimp (*Artemia *ssp.) toxicity test [13]. Moreover, four compounds isolated from *C. albida* exhibited cytotoxic activities against the tumor KB cell line at concentrations of 7–19 *μ*g/mL [13].

Finally, the ability of HEE and MEE from *L. confusa* to decrease the inflammatory response elicited by LPS is in agreement with other anti-inflammatory activities reported for *L. sophiifolia* in a carrageenan-induced paw oedema in mice [12].

## 5. Conclusion

The extracts and fractions from the plant species *L. confusa* represent a new, valuable source of antimicrobial agents against bacterial, fungal, and parasitic pathogens. Of special interest is the potency of two fractions from the CEE (CE 4) and MEE (ME 3), which show strong anti-MRSA activity. Moreover, three fractions from the HEE (HE 3, HE 4, HE 8) show antimycotic activity against the pathogens *C. neoformans* and *T. rubrum*. We also show that AEE and CEE, as well as the chloroformic fraction CE 2, were effective as antileishmanial agents, and HEE and MEE significantly reduce the secretion of IL-6, a cytokine involved in the progression of inflammatory processes.

Although, in its native Mexican growth area, *L. confusa* is only used in folk medicine as a sedative and to treat addictions, in this paper we provide evidence that *L. confusa* possesses antimicrobial, antiparasitic, and anti-inflammatory bioactivities similar to those previously reported for the genus *Conyza*. Our study supports the fact that this plant can potentially be used for the treatment of other illnesses outside its current use in folk medicine. Although the extracts and fractions obtained from this plant have shown cytotoxic effects against human cells, it could still be useful in topical formulations for the treatment of skin infection caused by *S. aureus *in both humans and animals. Moreover, although this work intended to screen the bioactivities using a cell line model, studies of the bioactivities using a murine model would be advantageous to demonstrate the bioactivities *in vivo*. Future research is required to identify and isolate the plant's bioactive compounds to assess its potential as a candidate for drug discovery programs.

## Figures and Tables

**Table 1:** Antimicrobial activity of extracts and fractions of *Laennecia confusa* expressed as MIC (*μ*g/mL).

	Bacteria					Fungi		
	EC	KP	MRSA	PA	SA	CA	CN	TR
HE 1	NA	NA	1000	NA	1000	NA	NA	NA
HE 3	NA	NA	1000	NA	1000	1000	NA	100
HE 4	NA	NA	NA	NA	NA	1000	200	30
HE 8	NA	NA	NA	NA	NA	1000	1000	100
CEE	NA	NA	NA	NA	1000	NA	NA	NA
CE 1	NA	NA	NA	NA	NA	1000	NA	NA
CE 2	NA	NA	NA	NA	NA	1000	NA	NA
CE 3	NA	NA	1000	NA	NA	100	NA	50
CE 4	NA	1000	200	NA	NA	1000	1000	NA
ME 3	NA	1000	200	NA	1000	NA	NA	NA
ME 4	1000	1000	1000	1000	NA	NA	NA	NA
ME 5	NA	NA	1000	NA	NA	NA	NA	NA

EC: *Escherichia coli*; KP: *Klebsiella pneumoniae*; MRSA: methicillin-resistant *Staphylococcus aureus*; PA: *Pseudomonas aeruginosa*; SA: *Staphylococcus aureus*; CA: *Candida albicans*; CN: *Cryptococcus neoformans*; TR: *Trichophyton rubrum*. NA: no activity detected; CEE: chloroform extract; HE: hexanic fractions; CE: chloroformic fractions; ME: methanolic fractions. Experiments were performed in triplicate.
